# Myeloid-Derived Suppressor Cells Restrain Natural Killer Cell Activity in Acute Coxsackievirus B3-Induced Myocarditis

**DOI:** 10.3390/v13050889

**Published:** 2021-05-12

**Authors:** Irene Müller, Lisa Janson, Martina Sauter, Kathleen Pappritz, Sophie Van Linthout, Carsten Tschöpe, Karin Klingel

**Affiliations:** 1 BIH Center for Regenerative Therapies (BCRT), Berlin Institute of Health at Charité-Universitätsmedizin Berlin, 10017 Berlin, Germany; imuellerweb@gmail.com (I.M.); kathleen.pappritz@charite.de (K.P.); sophie.van-linthout@charite.de (S.V.L.); carsten.tschoepe@charite.de (C.T.); 2 DZHK (German Center for Cardiovascular Research), Partner Site Berlin, 10017 Berlin, Germany; 3 Cardiopathology, Institute for Pathology and Neuropathology, University Hospital Tübingen, 72076 Tübingen, Germany; janson.lisa@gmx.de (L.J.); Martina.Sauter@med.uni-tuebingen.de (M.S.); 4 Department of Cardiology, Campus Virchow Clinic, Charité-Universitätsmedizin Berlin, 10017 Berlin, Germany

**Keywords:** myeloid-derived suppressor cells, myocarditis, coxsackievirus B3, natural killer cells

## Abstract

Murine models of coxsackievirus B3 (CVB3)-induced myocarditis well represent the different outcomes of this inflammatory heart disease. Previously, we found that CVB3-infected A.BY/SnJ mice, susceptible for severe acute and chronic myocarditis, have lower natural killer (NK) cell levels than C57BL/6 mice, with mild acute myocarditis. There is evidence that myeloid-derived suppressor cells (MDSC) may inhibit NK cells, influencing the course of myocarditis. To investigate the MDSC/NK interrelationship in acute myocarditis, we used CVB3-infected A.BY/SnJ mice. Compared to non-infected mice, we found increased cell numbers of MDSC in the spleen and heart of CVB3-infected A.BY/SnJ mice. In parallel, S100A8 and S100A9 were increased in the heart, spleen, and especially in splenic MDSC cells compared to non-infected mice. In vitro experiments provided evidence that MDSC disrupt cytotoxic NK cell function upon co-culturing with MDSC. MDSC-specific depletion by an anti-Ly6G antibody led to a significant reduction in the virus load and injury in hearts of infected animals. The decreased cardiac damage in MDSC-depleted mice was associated with fewer Mac3^+^ macrophages and CD3^+^ T lymphocytes and a reduced cardiac expression of S100A8, S100A9, IL-1β, IL-6, and TNF-α. In conclusion, impairment of functional NK cells by MDSC promotes the development of chronic CVB3 myocarditis in A.BY/SnJ mice.

## 1. Introduction

Myocarditis is defined as an inflammatory cardiac disorder mostly caused by viral infections and represents a main cause of heart failure in young adults [1]. Despite classical pharmacological heart failure treatment, approximately 30% of acute myocarditis can progress to a chronic dilated phenotype (inflammatory dilated cardiomyopathy) with an impaired prognosis. This emphasizes the need to understand the mechanisms determining the course of myocarditis in view of finding novel therapeutic options [2]. There is evidence that besides the direct viral-induced cardiocytotoxic effects, the immune system and thus the host genetics play a central role in the course of myocarditis [3,4]. It was previously shown that coxsackievirus B3 (CVB3)-infected susceptible mice differ from mice being resistant to chronic myocarditis in their natural killer (NK) cells’ maturation profile, function, and activation [5]. There is evidence that besides the already known inhibitory effect of myeloid-derived suppressor cells (MDSC) on T lymphocytes [6], MDSC are also involved in the inhibition of NK cells [7]. The exact mechanisms by which MDSC mediate suppression of NK cells are unknown [8]. In general, markers for NK-induced cytotoxic responsiveness to virus-infected cells include granzyme B (GzmB) and CD107a and the receptor Natural-killer group 2 member D (NKG2D). NKG2D was found to be relevant for the different outcomes of myocarditis in vivo comparing A.BY/SnJ mice, being susceptible to chronic myocarditis, and C57BL/6J mice, being resistant to chronic myocarditis [5]. Furthermore, NK cells are known to produce pro-inflammatory markers such as TNF-α and MIP-1α, promoting immune cell infiltration [9]. Regarding MDSC, it was previously described that immature bone marrow myeloid cells are transforming to MDSC instead of matured macrophages, granulocytes, and dendritic cells under pathophysiological conditions, such as cancer [10,11]. Basically, MDSC are divided in granulocytic (CD11b^+^Ly6G^high^ Ly6C^low^)- and monocytic (CD11b^+^Ly6G^low^Ly6C^high^)-positive cells [12]. Furthermore, there is evidence that CVB3-induced myocarditis is influenced by MDSC [13,14], which are beside neutrophils and monocytes, the main source of the pro-inflammatory alarmins S100A8 and S100A9, which mainly appear as a heterodimer S100A8/9 [15]. The accumulation and migration of MDSC are largely regulated by S100A8/9 [15].

The aim of this study was to investigate whether MDSC play a role in the outcome of myocarditis by evaluating cardiac damage, virus load, and the immune cell response in susceptible A.BY/SnJ mice. Importantly, we identified the involved pathomechanisms by the depletion of MDSC in vivo and in cell culture experiments, where we aimed to evaluate the reaction of NK cells in CVB3-infected co-cultured HeLa/RAW (macrophages) cells in the presence or absence of MDSC.

## 2. Materials and Methods

### 2.1. In Vivo Experiments

A.BY/SnJ (H-2b) mice were purchased from Charles River (Bar Harbor, ME, USA). Male mice were used for experiments at the age of 4–5 weeks. Experiments were conducted according to the German animal protection law with the permission of the Regierungspräsidium Tübingen, Germany (Permit No PA2/10). CVB3 used in this study was derived from the infectious cDNA copy of the cardiotropic Nancy strain [5]. Mice were infected intraperitoneally (ip) with 1 × 10^5^ plaque forming units (pfu) of purified CVB3. At various time points after infection (0, 4, and 8 days post infection (dpi)), mice were sacrificed, and hearts and spleens were collected and analyzed.

### 2.2. In Vivo Depletion of MDSC

A.BY/SnJ mice received ip 150 μg of the anti-Ly6G (Clone RB6-8C5) antibody (BioXCell, Köln, Germany) one day before infection (-1), as well as on days 1 and 3 post infection (pi). The antibody binds as a clone of RB6-8C5 to Ly6G as well as to Ly6C and depletes the complete Gr-1 cell population [12]. Confirmation of depleted Gr-1 cells in the spleen was proven in control mice and mice 4 dpi by flow cytometry.

### 2.3. Isolation of Splenic and Cardiac Immune Cells

Mononuclear cells from spleens were isolated by Ficoll-Histopaque (Sigma Aldrich, Munich, Germany) density gradient centrifugation as described elsewhere [5]. For purification of single cell suspension from hearts, the myocardium was digested with collagenase II (Genaxxon, Ulm, Germany). Released cells were separated by passing through a cell strainer (100 μm), and then isolated by Ficoll-Histopaque density gradient centrifugation.

### 2.4. Enrichment of Splenic NK and MDSC for In Vitro Experiments

Splenic immune cells were isolated as described above. Enrichment of NK cells from non-infected A.BY/SnJ mice and of MDSC from CVB3-infected A.BY/SnJ mice (4 dpi) was performed via Magnetic Activated Cell Sorting (MACS, Miltenyi, Bergisch Gladbach, Germany). For NK cells, the NK Isolation Kit II (Miltenyi, Bergisch Gladbach, Germany) was used and for MDSC, the MDSC Isolation Kit (Miltenyi, Bergisch Gladbach, Germany) was used. Isolated cells were used for in vitro experiments as described below.

### 2.5. Flow Cytometry

For flow cytometry, 1 × 105 cells were incubated with the following antibodies: for MDSC identification: Ly6G (1A8)-FITC (BioLegend, Koblenz, Germany), anti-CD11b (M1/70)-PE, and anti-Ly6C (HK1.4)-APC (both Miltenyi, Bergisch Gladbach, Germany). For S100A8/A9 identification on splenic CD11b^+^ Gr-1^+^ cells, we used rabbit-anti-mouse-S100A8/rabbit-anti-mouse-S100A9, goat-anti-rabbit-AF647, anti-Gr-1 (RB6-8C5)-FITC, anti-CD11b (M1/70)-PE antibodies. For confirmation of splenic MDSC depletion, the anti-Gr-1 antibody (RB6-8C5)-FITC (Miltenyi, Bergisch Gladbach, Germany) was used. For in vitro experiments, the anti-GzmB (GB11)-AlexaFluor 647, anti-NKG2D (CX5)-rat and goat-anti-rat-AF647, and anti-NKp46 (29A1)-FITC antibodies were used. Cells were analyzed on a FACSCalibur (BD Bioscience, Heidelberg, Germany), collecting 100,000 events in a live gate. The data were analyzed using the FlowJo software 8.8.8 (Tree Star Inc, Ashland, OR, USA).

### 2.6. RNA Isolation, cDNA Synthesis, and RT-PCR for the Detection of Cytokines and Viral RNA

Total RNA of frozen heart and spleen tissue was isolated using the TriFast-reagent (Peqlab, Erlangen, Germany) according to the manufacturer’s instructions. Isolated RNA (0.2 µg) was used to perform one-step quantitative real-time RT-PCR (TaqMan One-Step RT-PCR Master Mix Reagents Kit, Life Technologies GmbH, Darmstadt, Germany) as described [16]. To assess the relative mRNA expression, RT-PCR was performed using gene expression assays, containing probes labelled with FAM at 5’ and with a quencher TAMRA at 3’, from Life Technologies. Data analysis of IL-1β, IL-6, MIP-1α, S100A8, and S100A9 was performed in relation to the expression of the housekeeping gene hypoxanthine-guanine-phosphoribosyltransferase (HPRT) as an internal standard. The number of CVB3 genomes was determined in relation to an external virus standard of known copy numbers (RNA transcripts — generated by in vitro transcription of a CVB3-R1 plasmid [17]) using PanEntero-primers and a probe as described [16]. Primers and probes were purchased from MWG-Biotech, Ebersberg, Germany: IL-1β: forward GTCGCTCAGGGTCACAAGAAA, reverse CATCAGAGGCAAGGAGGAAAAC, probe FAM-CATGGCACATTCTGTTCAAAGAGAGCCTG-TAMRA; IL-6: forward ACAAGTCGGA GGCTTAATTACACAT, reverse AATCAGAATTGCCATTGCACAA, probe FAM-TTCTCTGG GAAATCGTGGAAATGAGAAAAGA-TAMRA; TNF-α: forward TCATGCACCACCA TCAAGGA, reverse TGCAGAACTCAGGAATGGACAT, probe FAM-AAATGGGCTTTCCG AATTCACTGGAGC-TAMRA; MIP-1α: forward GCGCCATATGGAGCTGACAC, reverse TCAGGCATTCAGTTCCAGGT, probe FAM-CCTGCTGCTTCTCCTACAGCCGGA-TAMRA; S100A8: forward TCCTTGCGATGGTGATAAAAGTG, reverse CCCAGCCCTAGGCCAGAA, probe FAM-TCTCACAAAGACAGCCACAAGGAGTAGCAGAG-TAMRA; S100A9: forward GCTTTGAGGAGTGTATGATGCTGAT, reverse TGGGTTGTTCTCATGCAGCTT, probe FAM-CAAAGTTGATCTTTGCCTGTCATG-TAMRA; HPRT: forward GACCGGTCCCGTCATG, reverse ACCTGGTTCATCATCGCTAA, probe FAM-CGACCCGCAGTCCCAGCG-TAMRA; PanEntero1: forward TCCTCCGGCCCCTGA, reverse RATTGTC-ACCATAAGCAGCCA, probe FAM-CGGAACCGACTACTTTGGGTGWCCGT-TAMRA.

### 2.7. Immunohistological Stainings

For immunohistochemistry, 5um paraffin tissue sections were pretreated with 10 mM citrate buffer, pH 6, for 5 min in a pressure cooker followed by incubation for 1 h at 25 °C with a rat anti-mouse antibody recognizing Mac-3 (BD Bioscience, clone M3/84, 1:300) on macrophages and with a rabbit anti-CD3 antibody recognizing CD3 (Thermo Scientific, clone SP7, 1:500) on T lymphocytes, we used rabbit-anti-S100A8 (polyclonal rabbit ab, Biomedia, Foster City, CA) and rabbit-anti-S100A9 (polyclonal rabbit ab, Biomedia, Foster City, CA), respectively. Controls using normal sera were run to exclude non-specific staining. Slides were processed using the MACH 4 Universal HRP Polymer (Biocare Medical, Pacheco, CA, USA) and HistoGreen (Linaris, Dossenheim, Germany) as substrate. Tissue sections were counterstained with hematoxylin and finally embedded in Entellan.

### 2.8. Immunofluorescence Staining of Cardiac MDSC

For immunofluorescence stainings, hearts were cut in 6 μm-thick sections. For stainings, specific antibodies were used including anti-CD11b (M1/70), AlexaFluor 488, anti-Gr-1 (RB6-8C5), AlexaFluor 647, anti-Ly6G (1A8), and AlexaFluor 647 (BioLegend, Koblenz, Germany) followed by DAPI (4′,6-Diamidin-2-phenylindol) staining. Evaluation of the immunofluorescence sections was performed with the program AxioVision (Zeiss, Oberkochen, Germany).

### 2.9. In Vitro Experiments to Evaluate Regulation of NK Cells by MDSC

We aimed to evaluate the reactions of NK cells on CVB3-infected co-cultured HeLa/RAW (macrophages) cells in the presence or absence of MDSC. In general, during viral infection, NK cells receive activating signals, not only from infected cells (in our case, CVB3-infected HeLa cells), but also from infiltrating immune cells, such as macrophages [18]. In order to simulate the in vivo situation, HeLa/RAW co-culture experiments were performed. One day before CVB3 infection, HeLa and RAW cells were co-cultured in a 24-well plate, each at a cell density of 2 × 10^4^ cells/well in a DMEM (high Glucose) medium supplemented with 10% FCS and 1% penicillin-streptomycin. Cells were incubated for 24 h at 37 °C and 5% CO2. One hour before infection, the DMEM medium was substituted by pure medium without supplements, followed by CVB3 infection at a multiplicity of infection (MOI) of 10. Enriched NK cells from non-infected isolated A.BY/SnJ mice were taken up in RPMI 1640 (Life Technologies) with mIL-2 (250 U/mL). Subsequently, these cells were added to non-infected or CVB3-infected Hela/RAW cell co-cultures (ratio of NK cells to HeLa cells: 10:1). Likewise, MDSC-incubated NK cells (ratio 1:1) were also incubated in RPMI 1640 with mIL-2 (250 U/mL), followed by supplementation to non-infected and CVB3-infected Hela/RAW cell co-cultures (ratio of NK cells to HeLa cells: 10:1). Three hours later, cells were incubated with Brefeldin A (0.02 μg/μL), a protein transporter inhibitor, which enables visualization of intracellular cytokines. Cells were stained with the appropriate markers (as mentioned above) for intracellular granzyme B, and surface markers CD107a and NKG2D, and were submitted to flow cytometry.

### 2.10. Statistical Analyses

The data were statistically analyzed with GraphPad Prism 9 (GraphPad Software, La Jolla, CA, USA). All data are expressed as the mean ± SEM. For the comparison of non-normally distributed data, a Kruskal-Wallis test with an uncorrected Dunn’s post hoc test was used, whereas for normally distributed data, the One-way ANOVA with Fisher’s LSD post hoc test was performed. *p* < 0.05 was considered statistically significant.

## 3. Results

### 3.1. Regulation of Myeloid-Derived Suppressor Cells in the Spleen and Heart of Coxsackievirus B3-Infected A.BY/SnJ Mice

**Figure 1:**
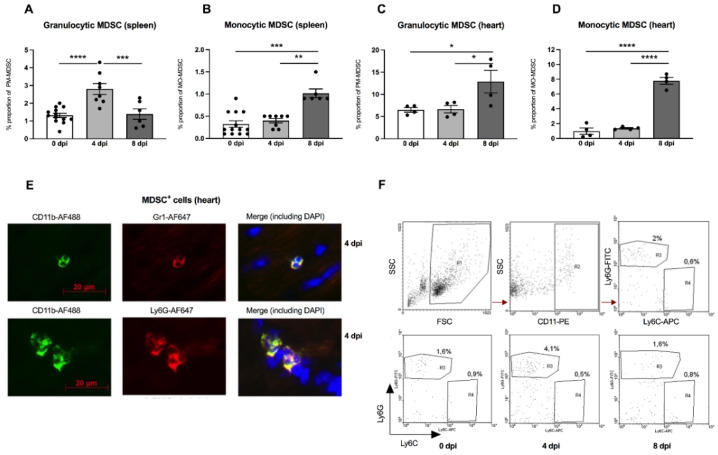
Granulocytic and monocytic MDSC subpopulations are increased in the spleen and heart of CVB3-infected A.BY/SnJ mice. Isolated splenic and cardiac immune cells were selected based on CD11b^+^Ly6GhighLy6Clow (granulocytic MDSC; (**A**,**C**)) and on CD11b^+^Ly6GlowLy6Chigh (monocytic MDSC; (**B**,**D**)). The y-axis displays the percentual proportion of 50,000 and 20,000 measured splenic or cardiac cells, respectively. The panel below (**E**) shows representative immunofluorescence pictures of cardiac tissue 4 days post infection (dpi) stained with anti-CD11b-AF488, anti-Gr1-AF647 (monocytic), anti-Ly6G-AF647 (granulocytic), and DAPI. Representative images of flow cytometry of the spleen from naive mice (upper panel) and from mice 0 dpi, 4 dpi, 8 dpi CVB3 (lower panel) are shown in (**F**). The gating strategy is depicted as R1 = vital cells, R2 = CD11b^+^ cells, R3 = Ly6Ghigh granulocytic MDSC, R4 = monocytic MDSC. Data are expressed as mean ± SEM and were analyzed with One-way ANOVA or Kruskal-Wallis test (* *p* < 0.05, ** *p* < 0.01, *** *p* < 0.001, **** *p* < 0.0001 with *n* = 13 for 0 dpi, *n* = 8 for 4 dpi, and *n* = 6 for 8 dpi for the splenic immune cells (**A**,**B**) and *n* = 4/group for the cardiac immune cells (**C**,**D**)).

There is evidence that the outcome of enteroviral myocarditis may be influenced by MDSC [13,14]. However, the exact role of MDSC in A.BY/SnJ mice, which are susceptible to severe acute myocarditis, is unknown. Interestingly, in our experiments, we found a 2.1-fold (*p* < 0.0001) increase in granulocytic MDSC 4 days post infection (dpi) versus 0 dpi in the spleen of A.BY/SnJ mice as analyzed by FACS. Later, in CVB3 infection (8 dpi), the amount of the granulocytic subpopulation decreased 2.0-fold (*p* = 0.0005) versus 4 dpi (Figure 1A). With respect to monocytic MDSC, A.BY/SnJ mice displayed a significant increase at 8 dpi versus 4 dpi (2.5-fold (*p* = 0.0066)) (Figure 1B). Quantification of cardiac granulocytic MDSC at 4 dpi showed no significant difference in susceptible CVB3-infected A.BY/SnJ mice versus naive animals, whereas at 8 dpi the levels increased 1.9-fold (*p* = 0.0207) versus 4 dpi (Figure 1C). With respect to cardiac monocytic MDSC, there was no significant difference in CVB3-infected mice at 0 dpi versus naive animals at 4 dpi, whereas at 8 dpi the levels increased 5.8-fold (*p* < 0.0001) versus 4 dpi (Figure 1D). Representative immunofluorescence images of CD11b^+^ Gr-1^+^ MDSC and granulocytic Ly6Ghigh MDSC in cardiac tissue of A.BY/SnJ mice 4 dpi are provided in Figure 1E. Typical images of flow cytometry of the spleen from naive mice (upper panel) and from mice 0 dpi, 4 dpi, 8 dpi CVB3 (lower panel) are shown in Figure 1F.

### 3.2. Impact of MDSC on NK Cells In Vitro

**Figure 2:**
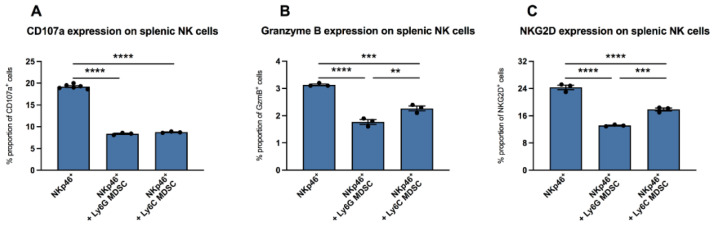
MDSC increased the expression of CD107a, granzyme B, and NKG2D in NK cells in vitro. Impact of Ly6G MDSC (granulocytic) or Ly6C MDSC (monocytic) on NK cells (NKp46+) in presence of CVB3-infected co-cultured HeLa/RAW cells. (**A**) Effect on the degranulation marker CD107a (**B**) granzyme B expression and (**C**) NKG2D receptor expression on NKp46^+^ NK cells. The y-axis displays the percentual proportion of 20,000 measured splenic NK cells. Data are expressed as mean ± SEM and were analyzed with One-way ANOVA or Kruskal-Wallis test (** *p* < 0.01, *** *p* < 0.001, **** *p* < 0.0001 with *n* = 3–6 for NKp46^+^, *n* = 3 for NKp46^+^+Ly6G MDSC, and *n* = 3 for NKp46^+^+Ly6C MDSC).

It is known that in vaccinia virus infection, MDSC are involved in the inhibition of functional NK cells [7]. Here, we wanted to evaluate whether there are also effects of MDSC on NK cells in CVB3 infection. We isolated NKp46^+^ splenic NK cells from uninfected mice and incubated them with CVB3-infected co-cultured RAW/Hela cells in the absence or presence of splenic MDSC from mice infected for four days. In the presence of granulocytic (Ly6G) MDSC as well as monocytic (Ly6C) MDSC, the NK surface marker CD107a was reduced by 2.3-fold (*p* < 0.0001) and 2.2-fold (*p* < 0.0001), versus unstimulated NK cells (NKp46^+^), respectively (Figure 2A). Further measurements revealed that culturing of NK cells with granulocytic MDSC as well as with monocytic MDSC also reduced the amount of granzyme B in NK cells by 1.8-fold (*p* < 0.0001) and 1.4-fold (*p* = 0.0002), respectively, versus control cells without MDSC (Figure 2B). A 1.9-fold (*p* < 0.0001) and 1.4-fold (*p* < 0.0001) reduction in NKG2D expression on NKp46^+^-cells was also recorded upon co-culture of NK cells with granulocytic MDSC as well as with monocytic MDSC versus untreated NK cells, respectively (Figure 2C).

### 3.3. Impact of MDSC Depletion on Cardiac Virus Load, Damage, and Immune Response in CVB3-Infected A.BY/SnJ Mice

**Figure 3:**
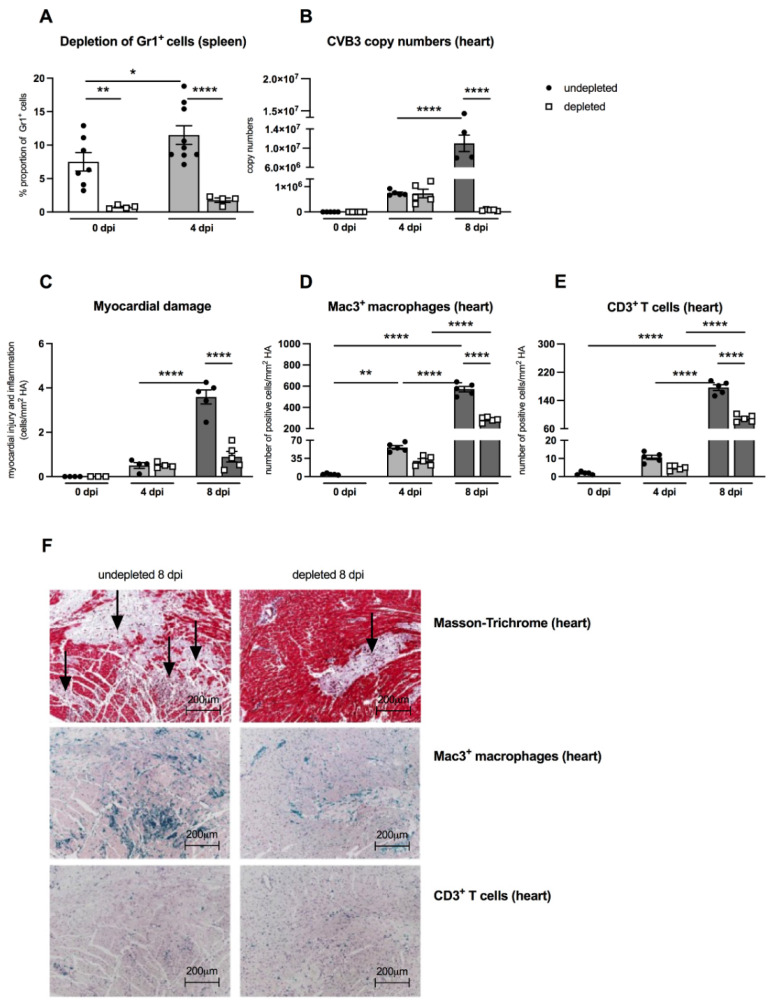
Gr-1 depletion decreases viral load, damage, and inflammation in the heart of CVB3-infected A.BY/SnJ mice. (**A**) Following treatment of CVB3-infected mice with anti-Ly6G (RB6-8C5), spleen cells were stained at 0 dpi and 4 dpi with an anti-Gr-1-FITC antibody and analyzed via flow cytometry. The y-axis displays the percentual proportion of 20,000 measured splenic cells (with *n* = 4–7 for 0 dpi and *n* = 4–9 for 4 dpi). (**B**) Next, hearts from Gr-1-depleted and non-depleted mice were analyzed regarding CVB3 copy number by quantitative real-time PCR at 0 dpi, 4 dpi, and 8 dpi (with *n* = 5 for 0 dpi, *n* = 5 for 4 dpi, and *n* = 4–5 for 8 dpi). (**C**) In addition, myocardial damage was quantitatively determined on hematoxylin-eosin (H/E) stained tissue sections at the respective time points (with *n* = 3–4 for 0 dpi, *n* = 4 for 4 dpi, and *n* = 5 for 8 dpi). Additionally, Gr-1 depletion decreases Mac3^+^ macrophages and CD3^+^ T lymphocytes in the heart of CVB3-infected A.BY/SnJ mice. After depletion of Gr-1^+^ cells with anti-Ly6G (RB6-8C5), the number of (**D**) Mac3^+^ macrophages and (**E**) CD3^+^ T cells/ mm^2^ heart area (HA) was quantified at 0, 4, and 8 dpi, using the respective immunohistological stainings. (**F**) Representative pictures display Masson-Trichrome staining (upper panel, arrow indicate damage), the immunohistological staining of Mac3^+^ cells (middle panel), and the staining of CD3^+^ cells (lower panel) of non-depleted and depleted animals at 8 dpi (magnification ×100). All data are expressed as mean ± SEM and were analyzed with One-way ANOVA or Kruskal-Wallis test (* *p* < 0.05, ** *p* < 0.01, **** *p* < 0.0001 with *n* = 5 for 0 dpi, *n* = 5 for 4 dpi, and *n* = 5 for 8 dpi).

An important aspect of the present work was to examine the effects of MDSC on the outcome of CVB3 myocarditis by comparing MDSC-depleted uninfected and infected A.BY/SnJ mice. The effectivity of depletion was controlled by flow cytometry. Figure 3A shows that in control animals as well as in CVB3-infected mice 4 dpi, the depletion led to 10.0-fold (*p* = 0.0042) and 6.5-fold (*p* < 0.0001) lower amounts of Gr-1^+^ cells in the spleen versus correspondent non-depleted mice, respectively. As viral replication plays a central role in the course of CVB3 myocarditis [19,20], we examined the effect of MDSC depletion on the CVB3 load via real-time RT-PCR. At 8 dpi, MDSC depletion resulted in a 157.0-fold (*p* < 0.0001) and 4.0-fold (*p* < 0.0001) diminished CVB3 RNA amount (Figure 3B) and cardiac damage (Figure 3C), respectively, versus non-treated mice.

As previously shown, the efficient viral replication in A.BY/SnJ mice is associated with a massive infiltration of immune cells into the heart [5]. Here, we evaluated the effect of MDSC depletion on cardiac inflammation via immunohistological quantification of Mac3^+^ macrophages and CD3^+^ T lymphocytes, which both account for the vast majority of immune cells in CVB3 myocarditis [21,22]. Eight dpi, MDSC-depleted A.BY/SnJ mice displayed 2.0-fold (*p* < 0.0001) and 2.0-fold (*p* < 0.0001) lower amounts of Mac-3^+^ macrophages (Figure 3D) and CD3^+^ T lymphocytes (Figure 3E), respectively, in cardiac tissue versus non-depleted infected mice. Representative immunohistological stainings obtained 8 dpi are shown in Figure 3F. Importantly, as shown in serial tissue sections, in the areas of inflammation, less cardiac foci of necrosis were found in depleted versus NON-depleted animals 8 dpi, as illustrated by Masson-Trichrome stainings (Figure 3F, upper panel; indicated by arrows).

### 3.4. Regulation of S100A8 and S100A9 in the Heart and Spleen

**Figure 4:**
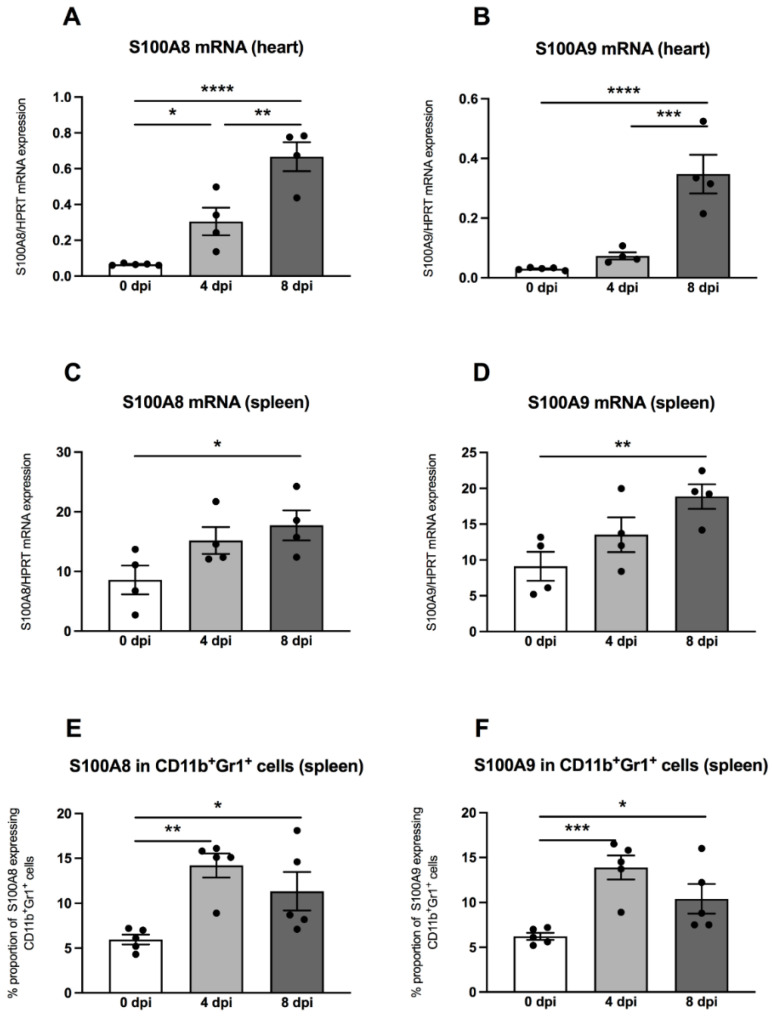
S100A8 and S100A9 mRNA and protein expression are increased in CVB3-infected A.BY/SnJ mice. mRNA levels of cardiac (**A**) S100A8, and (**B**) S100A9, and splenic (**C**) S100A8 and (**D**) S100A9 were measured by quantitative real-time PCR at 0, 4, and 8 dpi (with *n* = 5 for 0 dpi, *n* = 4 for 4 dpi, and *n* = 4 for 8 dpi) for the cardiac gene expression levels (**A**,**B**) and *n* = 4/group for the splenic gene expression levels (**C**,**D**). (**E**) S100A8 and (**F**) S100A9 protein expression in splenic CD11b^+^Gr-1^+^ cells measured by flow cytometry (*n* = 5). The y-axis displays the percentual proportion of 20,000 measured cardiac and splenic cells. Data are expressed as mean ± SEM and were analyzed with One-way ANOVA or Kruskal-Wallis test (* *p* < 0.05, ** *p* < 0.01, *** *p* < 0.001, **** *p* < 0.0001).

Previously, it was shown that MDSC are a main source of the S100A8 and S100A9 alarmins [15], which inspired us to measure these pro-inflammatory proteins in hearts and spleens by means of quantitative RT-PCR. In the hearts of A.BY/SnJ mice, there was a 4.7-fold (*p* = 0.0153) increase of S100A8 mRNA levels 4 dpi versus uninfected control animals (Figure 4A). Furthermore, a comparison of 4 versus 8 dpi revealed a further 2.2-fold (*p* = 0.0018) increase of S100A8 (Figure 4A). These findings were comparable for S100A9, which increased by 2.4-fold (*p* = 0.3927) at 4 dpi in cardiac tissue of A.BY/SnJ mice versus control animals and by 4.7-fold (*p* = 0.0003) at 8 dpi versus 4 dpi (Figure 4B). In spleens, at 4 dpi, S100A8 was increased but not significantly versus control animals (Figure 4C). At 8 dpi, spleens showed no further significant increase of S100A8 mRNA expression versus 4 dpi (Figure 4C). Similar observations were made for S100A9 (Figure 4D). Furthermore, we analyzed the S100A8 and S100A9 protein expression on splenic MDSC by flow cytometry. Four dpi, S100A8- and S100A9-positive MDSC were 2.4-fold (*p* = 0.0023) and 2.2-fold (*p* = 0.0009) higher, respectively, versus appropriate uninfected control mice (Figure 4E,F). There were no significant differences when S100A8- and S100A9-positive MDSC were compared 4 and 8 dpi. (Figure 4E,F).

### 3.5. Immunohistological Findings of Cardiac S100A8 and S100A9 Expressing Immune Cells

**Figure 5:**
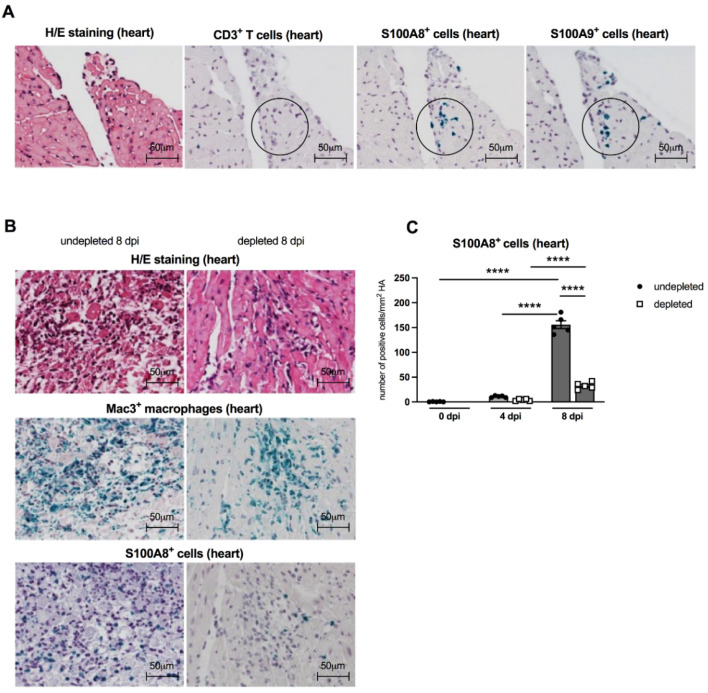
Cardiac S100A8- and S100A9-expressing cells are present before CD3^+^ T lymphocyte infiltration, and Gr-1 depletion results in a decrease of S100A8 protein levels in the heart of CVB3-infected A.BY/SnJ mice. (**A**) Representative H/E and immunohistological stainings for CD3^+^ T lymphocytes, S100A8, and S100A9 (see circles for positive green cells) in serial heart tissue sections of CVB3-infected mice 4 dpi (magnification ×400). Hearts from CVB3-infected mice with or without Gr-1-depletion were subjected to (**B**) H/E (upper panel), and immunohistochemical stainings of Mac3^+^ macrophages (middle panel, green cells) and S100A8 protein (lower panel, green cells) 8 dpi (magnification x400). (**C**) Subsequent quantification of S100A8 protein expression as number of positive cells/mm^2^ HA. Data are expressed as mean ± SEM and were analyzed with One-way ANOVA (**** *p* < 0.0001 with *n* = 5 for 0 dpi, *n* = 5 for 4 dpi, and *n* = 5 for 8 dpi).

In order to evaluate the patterns of S100A8- and S100A9-positive infiltrating cells in CVB3-infected A.BY/SnJ mice, we performed immunohistological stainings on serial sections of cardiac tissue. S100A8- and S100A9-expressing cells (see circles) were already present 4 dpi before CD3^+^ T lymphocytes (see circle) infiltrate the cardiac tissue [21], suggesting that not T cells but rather MDSC are the major S100A8 and S100A9 producers early in infection (Figure 5A). In cardiac tissue, fewer S100A8^+^ immune cells were detected in Gr-1-depleted CVB3-infected mice 8 dpi in comparison to non-depleted animals. Corresponding findings were observed for Mac3^+^ positive macrophages in consecutive heart tissue sections (Figure 5B). Findings in H/E stainings confirmed that Gr-1-depleted infected animals had much less cardiac necrosis and infiltrating cells versus CVB3-infected animals without depletion (Figure 5B). MDSC depletion resulted in a 4.8-fold (*p* < 0.0001) reduced number of S100A8-expressing cells at 8 dpi versus non-depleted infected mice (Figure 5C).

### 3.6. Impact of MDSC Depletion on Cytokine Levels

**Figure 6:**
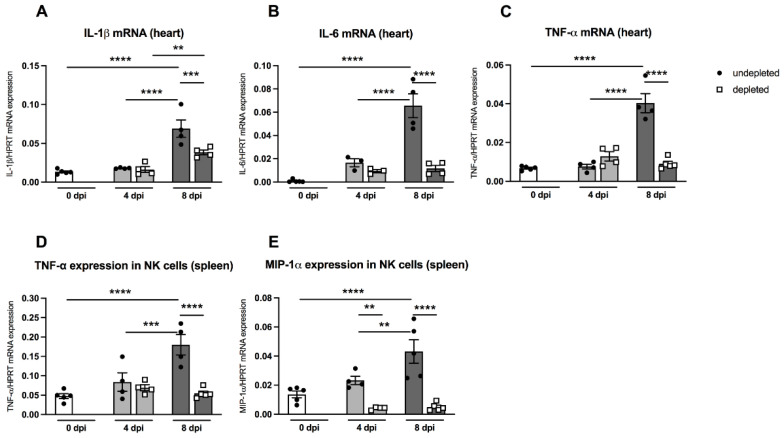
Gr-1 depletion decreases cardiac IL-1β, IL-6, and TNF-α mRNA levels in CVB3-infected A.BY/SnJ mice. Hearts of Gr-1-depleted and non-depleted CVB3-infected mice were subjected to (**A**) IL-1β, (**B**) IL-6, and (**C**) TNF-α mRNA quantification by real-time RT-PCR at 0, 4, and 8 dpi. Furthermore, Gr-1 depletion decreases TNF-α, and MIP-1α mRNA levels in splenic NK cells of CVB3-infected A.BY/SnJ mice. In detail, mRNA levels were quantified in splenic NK cells 0, 4, and 8 dpi of (**D**) TNF-α and (**E**) MIP-1α after Gr-1 depletion. Data are expressed as mean ± SEM and were analyzed with One-way ANOVA or Kruskal-Wallis test (** *p* < 0.01, *** *p* < 0.001, **** *p* < 0.0001 with *n* = 5 for 0 dpi, *n* = 3–4 for 4 dpi, and *n* = 4–5 for 8 dpi).

Pro-inflammatory cytokines such as IL-1β and IL-6 play an important role in CVB3-induced cardiac inflammation [23]. Furthermore, the cytokine TNF-α is known to block the maturation of myeloid precursor cells and thus enhances the amount of MDSC via S100A8/A9 production [24]. MDSC-depleted A.BY/SnJ mice displayed no significant changes in the heart regarding IL-1β, IL-6, and TNF-α mRNA levels 4 dpi versus non-depleted infected animals (Figure 6A). However, at 8 dpi, depletion of MDSC led to a 1.8-fold (*p* = 0.0009), 5.6-fold (*p* < 0.0001), and 4.4-fold (*p* < 0.0001) reduction in IL-1β, IL-6, and TNF-α compared to non-depleted infected animals (Figure 6A–C).

In order to investigate the in vivo effect of MDSC depletion on the function of NK cells, we determined the mRNA levels of the cytokines TNF-α and MIP-1α, which are known to be largely produced by NK cells, thus promoting the increased migration of immune cells into the heart [25,26]. As shown in Figure 6D and 6E, splenic NK cells from A.BY/SnJ mice displayed significantly enhanced levels of TNF-α mRNA 8 dpi compared to the levels of day 0 dpi (*p* < 0.0001) and 4 dpi (*p* < 0.001). Depletion of MDSC significantly reduced TNF-α mRNA expression in NK cells 8 dpi (*p* < 0.0001). Likewise, splenic NK cells revealed an increased production of MIP-α mRNA 8 dpi compared to day 0 dpi (*p* < 0.0001) and 4 dpi (*p* < 0.001). Following MDSC depletion, MIP-1α mRNA expression in NK cells was significantly reduced at 4 dpi (*p* < 0.01) and 8 dpi (*p* < 0.0001).

## 4. Discussion

Enteroviruses including CVB3 belong to the main aetiopathogenic agents of myocarditis [20]. There is firm evidence that the course of a CVB3 infection not only depends on the direct virus-induced cardiac damage, but also on the immune status, gender, and age of the host [3,4]. In mouse experiments, it was previously shown that the host genetics determine viral clearance and a subsequent healing or, on the other hand, viral persistence leading to chronic myocarditis [21,22]. It is well known that CVB3-infected susceptible A.BY/SnJ mice (H-2b) have, in comparison to resistant C57BL/6J mice (H-2b), a higher viral load and cardiac inflammation in the acute phase of infection [27,28]. In this context, we demonstrated that CVB3-infected susceptible A.BY/SnJ mice differ from C57BL/6J mice, being resistant to chronic myocarditis, in their NK cells maturation profile, function, and activation [5]. It was previously proven that MDSC are involved in the inhibition of NK cells [7], and their relevance was confirmed in experimental CVB3-induced myocarditis [13,14]. However, the role of MDSC and their relationship to NK cell activity in CVB3-induced myocarditis is largely unknown. As the total numbers of cardiac MDSC and S100A8/9-expressing MDSC as well as cardiac IL1-ß and IL-6 mRNA levels and inflammatory cells were significantly higher in ABY/SnJ mice than in resistant C57BL/6 mice, we concentrated on the role of MDSC in the outcome of myocarditis in permissive ABY/SnJ mice.

The findings of the current study demonstrate that MDSC influence the ability to eliminate the virus from the heart, which consequently leads to chronic myocardial inflammation [22,29]. In CVB3 myocarditis, NK cells play a central role as an early viral defense mechanism [5]. Markers for NK-induced cytotoxic responsiveness to virus-infected cells include granzyme B and CD107a, and the receptor NKG2D, for which an essential role was previously demonstrated comparing the outcome of CVB3-infection in A.BY/SnJ and C57BL/6J mice [5]. Here, we isolated splenic NK cells from uninfected mice, which were subjected to in vitro experiments with CVB3-infected co-cultured RAW/Hela cells in the absence or presence of splenic MDSC, in order to simulate the in vivo situation, where also macrophages function as NK-activating cells [18]. In the presence of granulocytic MDSC as well as monocytic MDSC, the reduction of granzyme B, CD107a, and NKG2D in splenic NK cells by MDSC point to a reduced cytotoxic ability of NK cells, which explains the reduced potential to clear the virus in A.BY/SnJ mice during the course of infection.

In order to evaluate how MDSC affect the outcome of CVB3 myocarditis in susceptible A.BY/SnJ mice, we next depleted MDSC in a CVB3 infection. We found that MDSC depletion led to a significant reduced cardiac virus load and injury compared to non-depleted CVB3-infected mice. These findings are in line with the study by Su et al. [13], who demonstrated that depletion of MDSC with an anti-Gr-1 antibody in CVB3-infected BALB/c mice significantly ameliorated the signs of myocarditis. Immune cells have protective properties and contribute to viral elimination in myocarditis [30]; however, an excessive activation of specific immune cells also influences the severity of cardiac damage [27]. In fact, we recorded a high number of T cells and macrophages in the hearts of CVB3-infected mice within areas of necrosis, whereas MDSC depletion resulted in a diminished cardiac immune cell infiltration and cardiac damage.

Regarding the activation, accumulation, and migration of MDSC, a particular emphasis is given to the pro-inflammatory mediators S100A8 and S100A9 [15]. We recently demonstrated that S100A8 and S100A9 play a pivotal role in human and experimental CVB3-induced myocarditis [31,32]. Several studies suggest a S100A8/9-induced autocrine feedback loop in MDSC, which abrogates the maturation of myeloid precursor cells, leading to an accumulation of MDSC [15]. The increased cardiac as well as splenic expression of S100A8 and S100A9 in CVB3-infected A.BY/SnJ mice and the chemotactic effect of the alarmins on MDSC [33] might explain the increased presence of MDSC in hearts of permissive mice. Similar findings were observed in Trypanosoma cruzi-infected mice, which displayed an enhanced cardiac expression of S100A8 and S100A9 upon infection in association with an increased presence of MDSC in the heart [34]. Furthermore, in a breast cancer mouse model, it was proven that MDSC not only express the receptor for S100A8/9 but also express these proteins themselves [33]. In the frame of the present study, flow cytometric analyses confirmed that MDSC express S100A8 and S1009 proteins in the course of acute CVB3 myocarditis. Immunohistological stainings provide evidence that rather MDSC and not T cells are the main S100A8 and S100A9 producers early in infection; the absence of MDSC resulted in a reduced cardiac expression of S100A8^+^ immune cells.

Findings in CVB3 mouse models suggest that the transition of the acute enterovirus myocarditis into a chronic phase depends on a cytokine-driven immune response [35], in which IL-1β plays a central role [23]. Correspondent to our observations in CVB3-infected mice [23], the murine encephalomyocarditis virus-induced myocarditis model also revealed an increased IL-1β expression, which correlated with a pronounced myocardial inflammation, damage, and fibrosis [36]. We and others have shown that the activation and accumulation of IL-1β also results in the induction of further immune modulators [23,37], such as IL-6 [38]. In mice, a cutaneous Staphyloccocus aureus infection was found to induce an increased expression of IL-6 and an enhanced amount of MDSC in the blood [39]. Thus, it is likely that upon MDSC depletion, the reduced cytokine production contributes to a reduced inflammatory cell infiltration, which in turn reduces cardiac damage.

NK cells, which are divided in different subpopulations, were previously shown to be protective in CVB3 myocarditis [5]. However, while one part of the NK cells acts in a cytotoxic manner via granzyme B and perforin to infected cells, the other part may promote immune cell infiltration by producing cytokines and chemokines such as TNF-α and MIP-1α, respectively [9]. In our in vivo experiments, we found increased TNF-α and MIP-1α mRNA levels in splenic NK cells of CVB3-infected mice, which decreased upon MDSC depletion. These data may be a hint that a NK cell-dependent attraction of pathogenic immune cells into the heart by pro-inflammatory cytokines is suppressed upon MDSC depletion, resulting in less inflammation. Our findings are supported by the observation of Cook et al. [40], who showed in CVB3-infected MIP-1α knock out mice comparably lower amounts of cardiac immune cells. In this context, it is important to mention that the cardiosplenic axis plays a decisive role in the outcome of myocarditis, as it is known that a pool of pro-inflammatory monocytes in the spleen is replenished and can be mobilized to the damaged heart [4]. However, the detailed characterization of splenic and cardiac MDSCs and their interaction is unknown and should be investigated in future studies.

In summary, we demonstrated in this study that MDSC interact with NK cells, determining the outcome of enteroviral myocarditis. Depletion of MDSC in CVB3-infected A.BY/SnJ mice resulted in a reduced virus load and cardiac damage, less pronounced inflammatory cell infiltration, and a lower expression of pro-inflammatory mediators, such as S100A8, S100A9, IL-1β, and IL-6. Moreover, we provided evidence that MDSC influence the cytotoxic activity of NK cells in enterovirus infection.

## Data Availability

The datasets used and/or analyzed during the current study are available from the corresponding author upon reasonable request.
